# Supplementary material for: Biodegradable silica nanoparticles for efficient linear DNA gene delivery
Source: Drug Deliv. 2024 Aug 5;31(1):2385376. doi: 10.1080/10717544.2024.2385376 (PMC11302475; doi:10.1080/10717544.2024.2385376)
Supplement: Supplemental Material [file IDRD_A_2385376_SM8612.pdf]

Supplementary Figures

Biodegradable silica nanoparticles for efficient linear DNA gene delivery

Andrés Ramos-Valle[1,2], Henning Kirst[1,2] , Mónica L. Fanarraga [1,2]

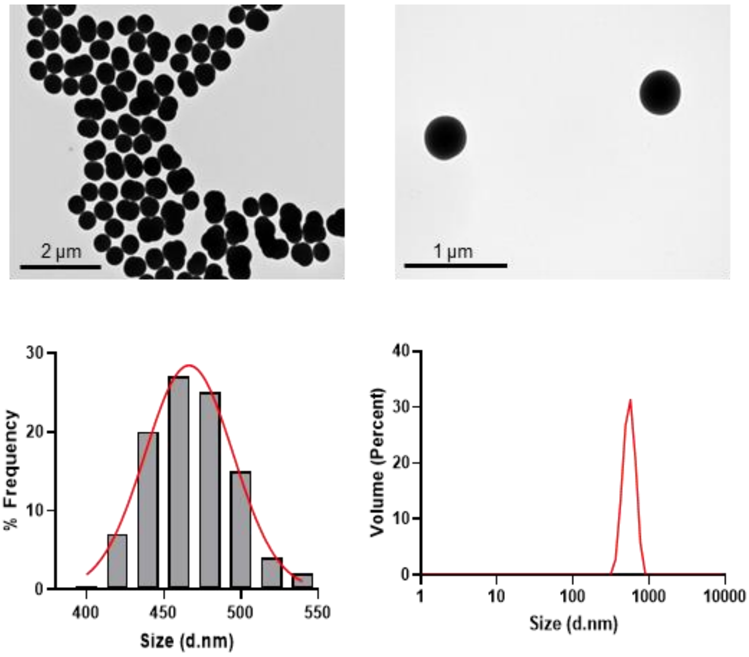

Figure S1. TEM characterization and DLS size distribution of p-DNA#1 particles.

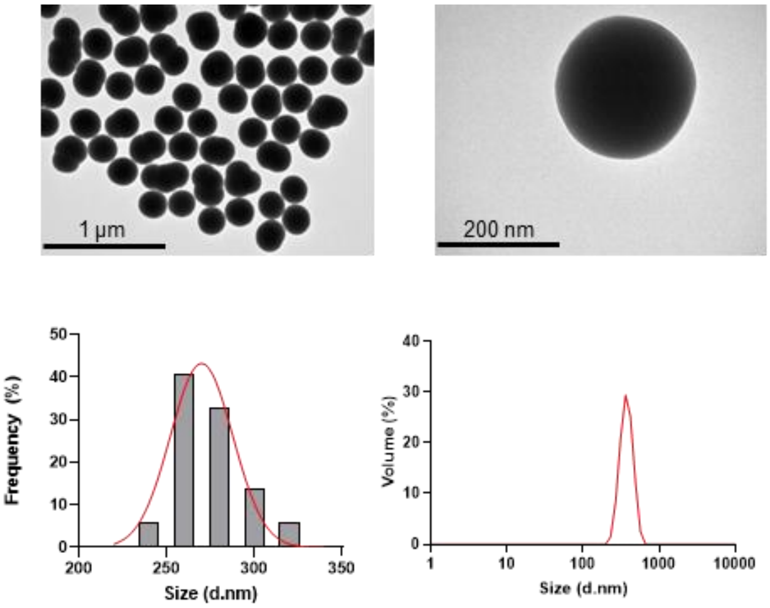

Figure S2. TEM characterization and DLS size distribution of p-DNA#2 particles.

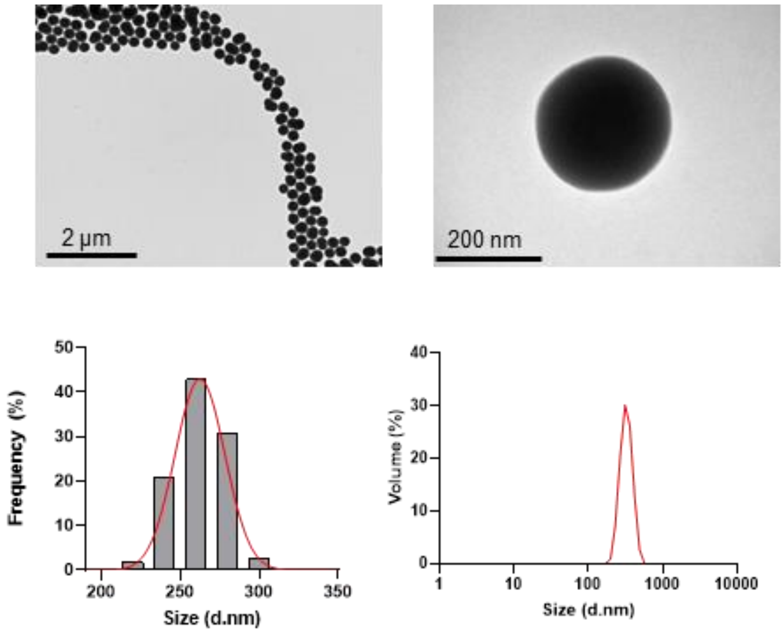

Figure S3. TEM characterization and DLS size distribution of p-DNA#3 particles.

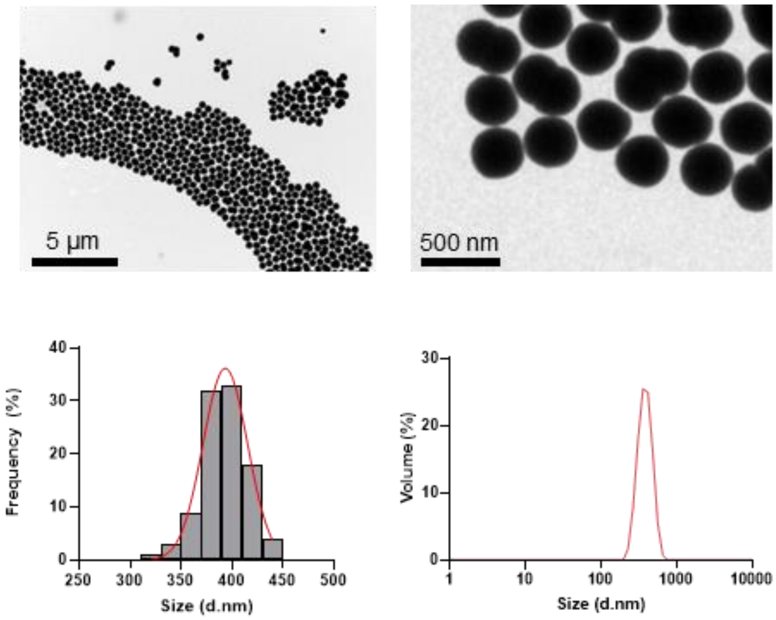

Figure S4. TEM characterization and DLS size distribution of p-DNA#4 particles.

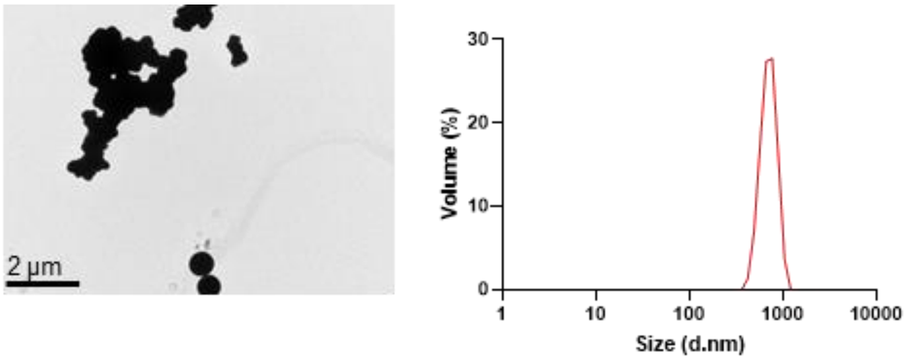

Figure S5. TEM characterization and DLS size distribution of p-DNA#5 particles.

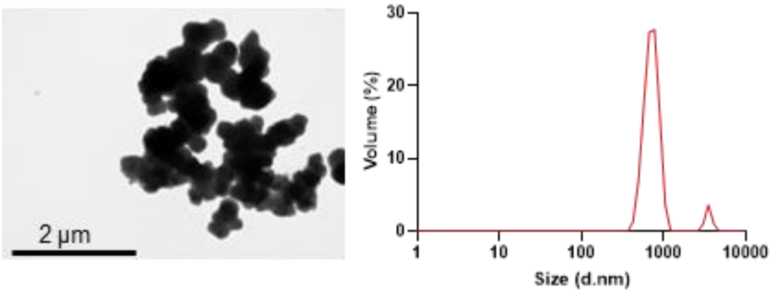

Figure S6. TEM characterization and DLS size distribution of p-DNA#6 particles.

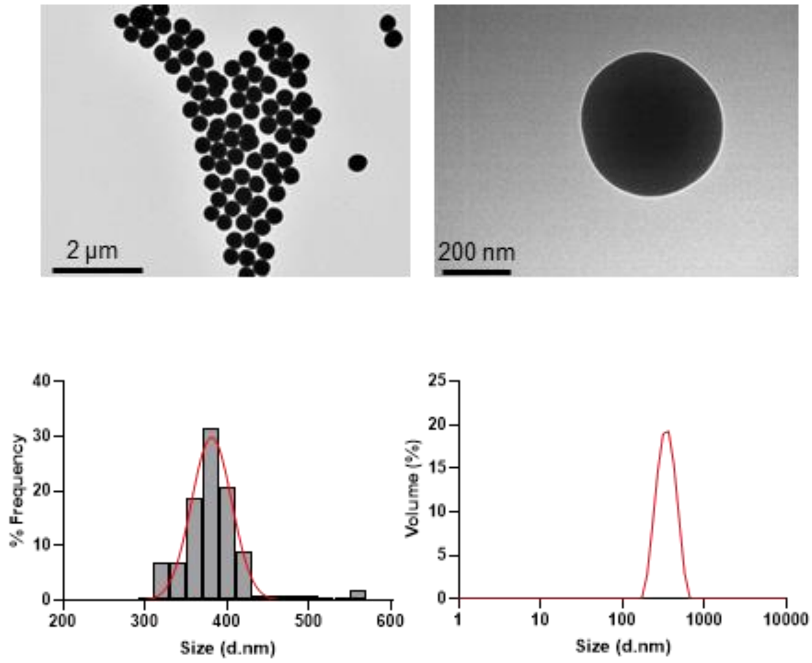

Figure S7. TEM characterization and DLS size distribution of l-DNA#1 particles.

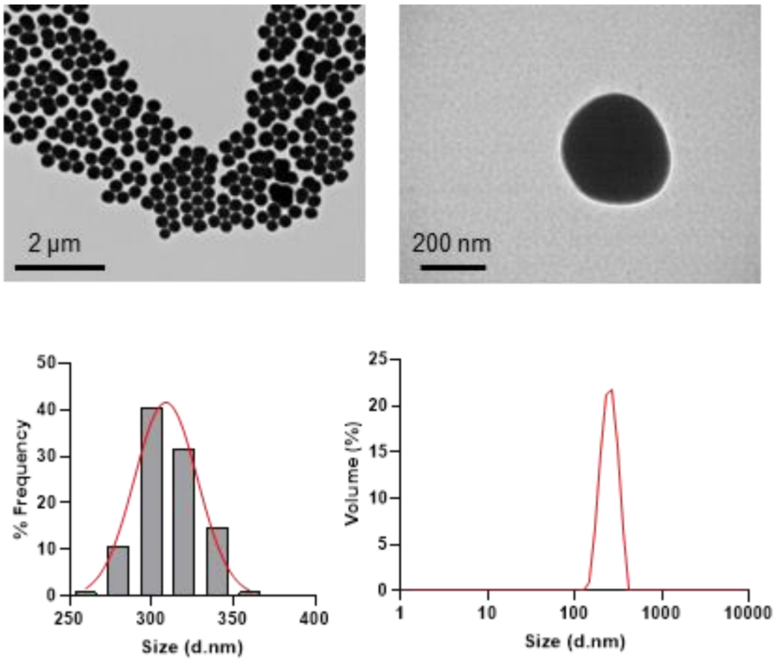

Figure S8. TEM characterization and DLS size distribution of l-DNA#2 particles.

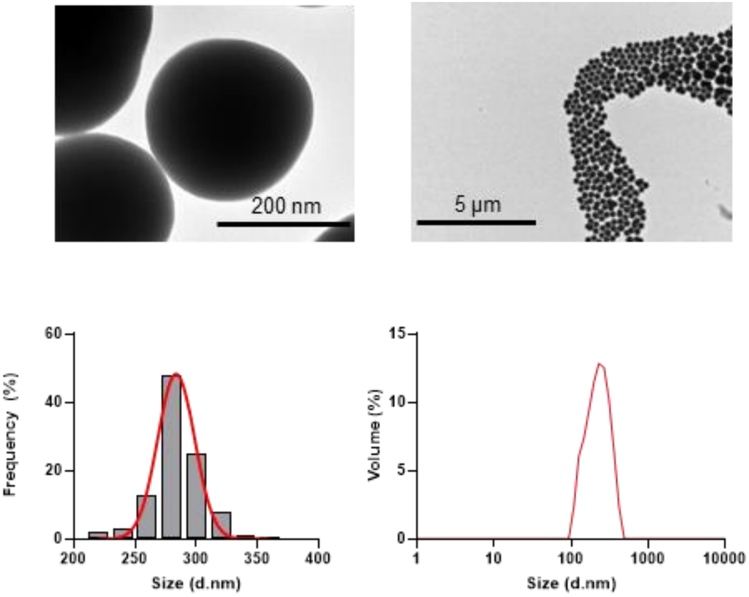

Figure S9. TEM characterization and DLS size distribution of l-DNA#3 particles.

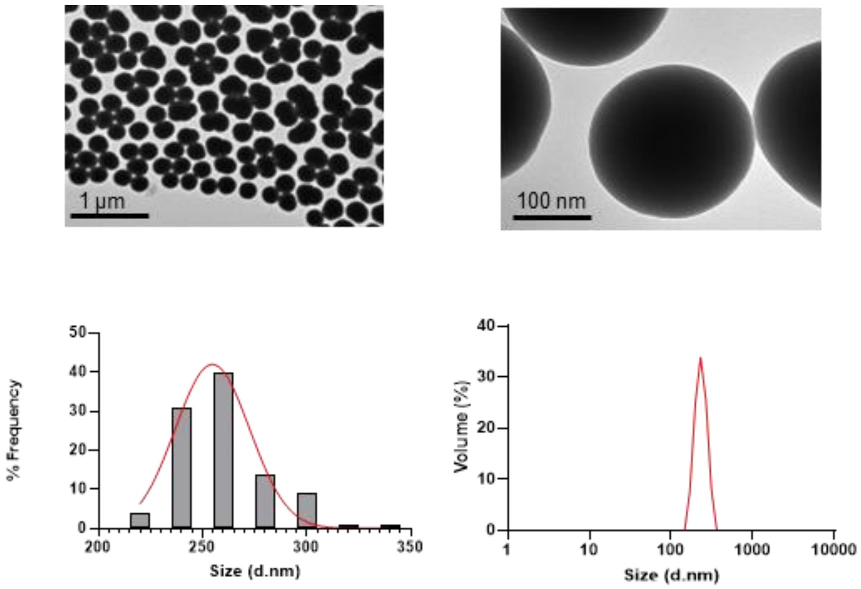

Figure S10. TEM characterization and DLS size distribution of I-DNA#4 particles.

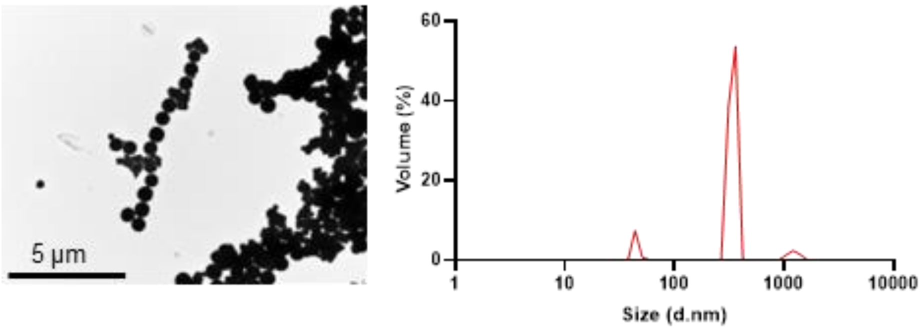

Figure S11. TEM characterization and DLS size distribution of l-DNA#5 particles.
